# Physicochemical, Structural, and In Vitro Gastrointestinal Tract Release Properties of Sodium Alginate-Based Cryogel Beads Filled with Hydroxypropyl Distarch Phosphate as a Curcumin Delivery System

**DOI:** 10.3390/molecules28010031

**Published:** 2022-12-21

**Authors:** Eun Chae Moon, Yoon Hyuk Chang

**Affiliations:** Department of Food and Nutrition and Bionanocomposite Research Center, Kyung Hee University, Seoul 02447, Republic of Korea

**Keywords:** cryogel beads, sodium alginate, hydroxypropyl distarch phosphate, encapsulation, curcumin delivery system, controlled-release

## Abstract

The objectives of this study were to produce sodium alginate (SA)-based cryogel beads filled with different concentrations (0, 0.4, 1.0, and 2.5%, *w*/*w*) of hydroxypropyl distarch phosphate (HDP) as a curcumin delivery system and to investigate the physicochemical, structural, and in vitro gastrointestinal tract release properties of the cryogel beads. According to FT-IR analysis, the formation of ionic crosslinking between SA and Ca^2+^ and the presence of HDP were found. XRD analysis demonstrated the successful encapsulation of curcumin in the beads by observing the disappearance of the characteristic peaks of curcumin. SEM analysis results revelated that SA-based cryogel beads exhibited a denser internal structure as the HDP concentration was increased. The encapsulation efficiency of curcumin in SA cryogel beads filled with HDP concentration from 0% to 2.5% was increased from 31.95% to 76.66%, respectively, indicating that HDP can be a suitable filler for the encapsulation of curcumin in the production of SA-based cryogel beads. After exposure to simulated gastric fluid (SGF) and simulated intestinal fluid (SIF), the release rate of curcumin was decreased as HDP concentration was increased. Accordingly, SA-based cryogel beads filled with HDP can be utilized for the delivery system of curcumin in the food industry.

## 1. Introduction

Cryogels can be considered as a type of hydrogel and have a highly interconnected and macroporous network which is formed by controlled polymerization at subzero temperatures [1]. Cryogelation is a recent technology that can form macroporous hydrogels ranging in various sizes from submicrometers to one hundred micrometers by controlling the pore size [2]. It has been well known that the structural properties of cryogels are considerably affected by several factors such as the chemical characteristics of precursors, the presence or the absence of fillers, their concentrations, and the time/temperature of cryogelation [3].

Curcumin is a naturally lipid-soluble polyphenolic compound obtained from turmeric (*Curcuma longa*) [4]. Recent studies have showed that curcumin has antioxidant, anti-inflammatory, and immunomodulatory properties, so that it is chosen as the target compound to be encapsulated [5,6,7]. However, the use of curcumin is limited in the food industry because of its chemical instability, rapid metabolism and poor bioavailability through oral intake. In particular, Di Meo et al. [8] noted that because the intestinal absorption of curcumin is relatively low, its oral bioavailability is low. Zhu et al. [9] reported that curcumin had a half-life of 10 min in a phosphate buffer with at pH > 7 because of the high instability in a neutral and alkaline environment. Even though curcumin is absorbed, it went through the fast metabolism in the liver and plasma. As a result, curcumin is considerably diverted into water-soluble metabolites including sulfate and glucuronide and passed out through urine [10]. Therefore, it is better for curcumin to reach the colon safely. Moreover, because of the low bioavailability of curcumin, it is frequently metabolized by colonic microorganisms such as *Lactobacillus*, *Bifidobacterium*, and *E. coli* [8]. According to Pluta et al. [11], the bidirectional interaction between curcumin and colonic microorganisms is as follows: the first interaction can be related to the direct regulation (microbial composition, diversity and richness) of colonic microorganisms by curcumin and the second interaction can be associated with the biotransformation of curcumin by colonic microorganisms, producing functional metabolites. The metabolic pathway of curcumin includes reduction, methylation, demethoxylation, hydroxylation, and acetylation by colonic microorganisms, and mainly tetrahydrocurcumin, dihydroferulic acid, and 1-(4-hydroxy-3-methoxyphenyl)-2-propanol are produced [12]. These metabolites of curcumin produced by colonic microorganisms possess characteristics and capabilities similar to curcumin. The metabolization of curcumin by colonic microorganisms can be responsible for various physiological benefits [12].

Sodium alginate (SA) is an anionic linear polysaccharide that can form three dimensional gels in the addition of divalent cations such as Ca^2+^ [13]. SA is normally used as a wall material for the encapsulation of bioactive compounds. However, the gel network obtained from SA is porous and very sensitive to harsh chemical environments, which leads to the rapid release of encapsulated bioactive compounds [14]. Therefore, it is hypothesized in this study that the addition of fillers can make an SA-based gel matrix with enhanced structural and physicochemical characteristics.

Hydroxypropyl distarch phosphate (HDP) is a resistant starch (RS 4 type) prepared by esterification with phosphorus oxide chloride or sodium trimetaphosphate and etherification with propylene oxide [15]. Resistant starch could not be degraded by digestive enzymes such as pancreatin and can be fermented by colonic microorganisms [16]. Therefore, it is hypothesized in the present study that HDP is an ideal filler for reducing the degradation of encapsulated curcumin in the gastrointestinal tract and delivering it to the colon. Furthermore, the previous literatures noted that HDP acts as a structural support to restrain the shrinkage during the drying process of SA beads [17].

There are a few previous studies on the production of SA-based delivery system adding native corn starch [18], and inulin [19]. However, no studies have reported the use of HDP as a filler for the preparation of SA-based cryogel beads, nor have studies been observed that elucidate the structural, physicochemical and in vitro release properties of SA-based cryogel beads filled with different concentrations of HDP as a curcumin delivery system. Therefore, the objectives of this study were (1) to produce SA-based cryogel beads filled with different concentrations of HDP as a curcumin delivery system, and (2) to evaluate the structural, physicochemical, and in vitro release properties of the cryogel beads.

## 2. Materials and Methods

### 2.1. Materials

Sodium alginate (SA) was purchased from Daejung chemicals, Siheung, Korea. Hydroxypropyl distarch phosphate (HDP) from waxy corn starch was provided from Samyangsa, Seoul, Korea. Calcium chloride (CaCl_2_) was purchased from ES FOOD, Gunpo, Korea. Curcumin (from *Curcuma longa*, powder, assay ≥ 80%), pepsin (from porcine gastric mucosa, ≥250 U/mg), and pancreatin (from porcine pancreas, 8 × USP specifications) were obtained from Sigma-Aldrich (St. Louis, MO, USA). The reagents and chemicals used in this study were of analytical grade.

### 2.2. Preparation of SA-Based Cryogel Beads Filled with HDP

According to the method of Lozano-Vazquez et al. [13] and Hu et al. [20] with slight modification, cryogel beads were prepared in this study. SA solution (1%, *w*/*w*) was prepared by dissolving SA in distilled water, stirring at 800 rpm for 30 min at 40 °C, and cooling at 800 rpm for 5 min at room temperature. Prior to adding curcumin into SA solution, and the curcumin stock solution was prepared by dissolving curcumin in 95% ethanol to 2.5 mg/mL. The curcumin stock solution (1 mL) and different concentrations (0, 0.4, 1.0, and 2.5%, *w*/*w*) of HDP were added to SA solution. The curcumin-SA-HDP mixture was stirred at 1000 rpm for 15 min to be completely dispersed. Thereafter, the hydrogel beads were formed by dripping the curcumin-SA-HDP mixture into 100 g of CaCl_2_ solution (3%, *w*/*w*) by using the syringe (22 G). Subsequently, 100 g of CaCl_2_ solution (3%, *w*/*w*) was additionally poured into the mixture to reinforce the crosslinking of hydrogel beads, and these were stirred at 400 rpm for 30 min. The hydrogel beads were obtained by vacuum filtration and rinsed with distilled water. The obtained hydrogel beads were first frozen at −18 °C for 18 h and thawed at 4 °C for 1 h. After the freeze-thaw cycle, they were rapidly frozen in a deep freezer at −80 °C and freeze dried to obtain cryogel beads using a lyophilizer (FD8508, Ilshin Biobase Co., Ltd., Dongducheon, Korea). Based on the different HDP concentrations, the cryogel beads filled with HDP (0, 0.4, 1.0, and 2.5%) were referred to as SA cryogel beads, SA-HDP_0.4_ cryogel beads, SA-HDP_1.0_ cryogel beads, and SA-HDP_2.5_ cryogel beads, respectively.

### 2.3. Scanning Electron Microscope (SEM) Analysis

The surface morphologies and microstructure of the cryogel beads were observed by a Scanning Electron Microscope (S-4700, Hitachi High-Technologies, Tokyo, Japan). The samples were studied at an accelerating voltage of 10 kV with 40 to 700 times magnification. All of the samples were lyophilized for 48 h before analysis.

### 2.4. X-ray Diffraction (XRD) Analysis

XRD patterns were recorded by X-ray diffractometer (D8 Advance., Bruker Co., Östliche Rheinbrückenstr, Germany) with a Cu Kα radiation (λ = 1.54060 Å). All samples were analyzed through the 2θ range from 5° to 50° at 3°/min at 40 kV and 40 mA. All samples were lyophilized for 48 h prior to analysis and analyzed by passing through a 180 μm mesh standard sieve.

### 2.5. Fourier Transform Infrared (FT-IR) Spectroscopy Analysis

FT-IR spectrum of the samples were measured by FT-IR Frontier (PerkinElmer, Frontier, USA) with an ATR sampling accessory. The IR spectrum in the range of 4000–500 cm^−1^ was recorded at 4 cm^−1^ resolution. All samples were lyophilized for 48 h prior to analysis and analyzed by passing through a 180 μm mesh standard sieve.

### 2.6. Differential Scanning Calorimetry (DSC) Analysis

DSC analyses were carried out using DSC LSM 800 (TA Instruments Inc., New castle, DE, USA). Each sample (1 mg) was placed onto a standard aluminum pan and heated from 30 °C to 320 °C at a heating rate of 10 °C/min under constant nitrogen purging at a flow rate of 50 mL/min. An empty sealed aluminum pan was used as a reference.

### 2.7. Encapsulation Efficiency of Curcumin

Encapsulation efficiency was measured using the method of Hu et al. [21] with slight modifications. One mL of 0.1 M trisodium citrate buffer (pH 6.0) was added to the curcumin encapsulated cryogel beads and stirred at 40 °C for 90 min to degrade the matrix. After all the matrix of cryogel beads was degraded, 0.8 mL of dimethyl sulfoxide (DMSO) was added to extract curcumin and stirred for 3 h. Next, the curcumin extract mixture was centrifuged at 3500 rpm for 10 min, and the supernatant was collected. The undissolved curcumin remaining in the sediment was dissolved again by adding 0.2 mL of DMSO and vortexed for 1 min. The mixture was centrifuged at 3500 rpm for 10 min, and the supernatant was collected. The total supernatant was used to determine the amount of encapsulated curcumin. The curcumin concentration (encapsulated amount of curcumin) in the cryogel beads was measured at 435 nm by a UV-Visible spectrophotometer (Thermo Scientific, Vantaa, Finland) with a calibration curve (0–10 μg/mL free curcumin). Measurements were performed in triplicate. The encapsulation efficiency was determined by using Equation (1), as shown below:

Encapsulation efficiency (%) = Encapsulated curcumin in samples/Total amount of added curcumin × 100(1)

### 2.8. Swelling Ratio

The experiment of swelling ratio was carried out according to Sáez et al. [22] with slight modifications. Cryogel beads (20 mg) were immersed in 20 mL of acidic medium (pH 1.2) and weakly alkaline medium (pH 7.4). They were then incubated in a shaking water bath under constant shaking (130 rpm) for 4 h at 37 °C. The swollen cryogel beads were filtered to remove excess medium with vacuum filtration and weighed immediately. The swelling ratio (g/g) was determined by the following Equation (2):

Swelling ratio (g/g) = W_t_/W_d_ (2)

where W_t_ and W_d_ are the weight of cryogel beads in swollen state at time t and the weight of cryogel beads before immersion state, respectively. All experiments were performed in triplicate.

### 2.9. In Vitro Gastrointestinal Tract Release Study

An in vitro gastrointestinal tract release study was determined with some modifications to the methods of the Yang & McClements [23] and Wu et al. [24] studies. The in vitro gastrointestinal tract release study of cryogel beads was carried out to investigate the controlled-release of the core material (curcumin) in simulated gastric fluid (SGF, pH 1.2) and simulated intestinal fluid (SIF, pH 7.4). SGF was prepared by dissolving 2 g of NaCl and 3.2 g pepsin in 1 L of distilled water and adjusting the pH to 1.2 using 1.0 M HCl. SIF was prepared by 1 mL of calcium chloride solution (83.25 mg/mL), 4 mL of bile salt solution (5 mg/mL) and 2.5 mL of pancreatin solution (4.8 mg/mL) in phosphate buffered saline (PBS) of pH 7.4.

Cryogel beads (100 mg) were immersed to 5 mL of SGF and SIF and incubated under constant shaking (130 rpm) in a shaking water bath at 37 °C for 2 h and 4 h, respectively. The withdrawn media (1 mL) was taken out at predetermined time intervals (after 1 and 2 h for SGF and 1, 2, and 4 h for SIF) and replaced with the same volume of fresh media. Then, 1 mL of withdrawn medium was mixed with DMSO for the spectrophotometric analysis described in Section 2.7. Measurements were performed in triplicate.

### 2.10. Statistical Analysis

All statistical analyses were performed using SAS version 9.4 (SAS Institute INC, Cary, NC, USA). An analysis of variance (ANOVA) was performed using the general linear models (GLM) procedure to determine significant differences among the samples. Means were compared by using Fisher’s least significant difference (LSD) procedure. Significance was defined at the 5% Level (*p* < 0.05).

## 3. Results and Discussion

### 3.1. Scanning Electron Microscope (SEM) Images

**Figure 1:**
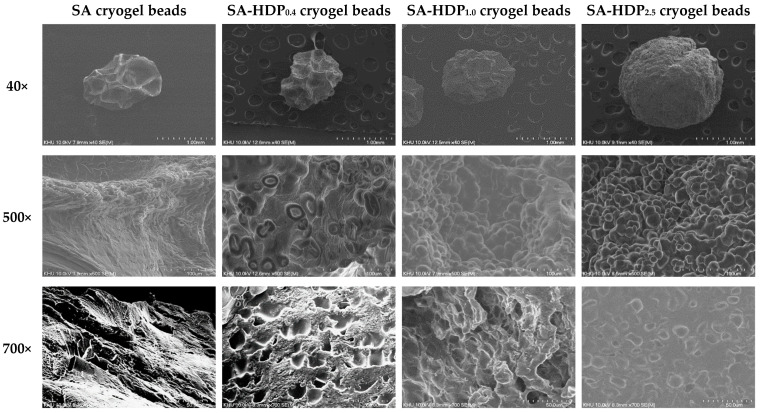
Scanning electron microscope (SEM) images of sodium alginate (SA)-based cryogel beads filled with different concentrations of hydroxypropyl distarch phosphate (HDP): overall morphologies (40×), surface morphologies (500×), cross-section morphologies (700×). SA cryogel beads, SA-HDP_0.4_ cryogel beads, SA-HDP_1.0_ cryogel beads, and SA-HDP_2.5_ cryogel beads represent cryogel beads filled with different concentrations of HDP (0, 0.4, 1.0, and 2.5%, respectively).

SEM images of SA cryogel beads, SA-HDP_0.4_ cryogel beads, SA-HDP_1.0_ cryogel beads, and SA-HDP_2.5_ cryogel beads are shown in Figure 1. The first row shows the overall morphology of the cryogel beads magnified by 40×, the middle row shows the surface morphology magnified by 500×, and the lowest row shows the surface of the cross-section morphology by 700× magnification.

The overall morphology (40×) of SA cryogel beads showed a non-spherical shape with subsidence. However, the surface subsidence was less apparent and became more spherical as the concentration of HDP was increased. Lyophilization causes sublimation of water crystals, which causes shrinkage of the cryogel matrix [25]. According to Zafeiri et al. [17], starch granules acted as structural support to restrain the shrinkage during the drying process of the alginate beads.

The surface morphology (500×) of SA cryogel beads showed cracks (red arrows). As the HDP concentration was increased, more HDP granules were embedded in the surface of the cryogel beads, resulting in a bumpy and rough surface. According to López Córdoba et al. [18], it was reported that corn starch granules were homogeneously distributed in the beads when starch was added to calcium alginate-starch hydrogels, confirming that starch granules were served as a filler. Therefore, it was clearly shown in this study that HDP granules acted as a filler in SA-based cryogel beads.

The cross-section morphology (700×) was suitable for observing the internal structure of the cryogel beads. Several large pores were identified in the internal structure of SA cryogel beads. However, the size and number of pores were decreased as the concentration of HDP was increased. In particular, in SA-HDP_2.5_ cryogel beads, the pore was not nearly observed because a large amount of HDP granules were embedded in the scaffold. Chan et al. [25] reported that the porous internal structure of calcium alginate beads was filled as corn starch (filler) was added, which was observed by SEM micrography and X-ray microtomography. Furthermore, adding resistant starch to oxidized gellan gum hydrogel beads made its internal structure denser [26].

Based on the results obtained from the overall, surface and cross-section morphology, as the concentration of HDP was increased, a spherical shape, a surface with embedded HDP granules, and a denser internal structure of the cryogel beads can be produced. These results indicated that HDP can be used as an effective filler to reinforce the SA-based cryogel beads matrix.

### 3.2. X-ray Diffraction (XRD) Analysis

**Figure 2:**
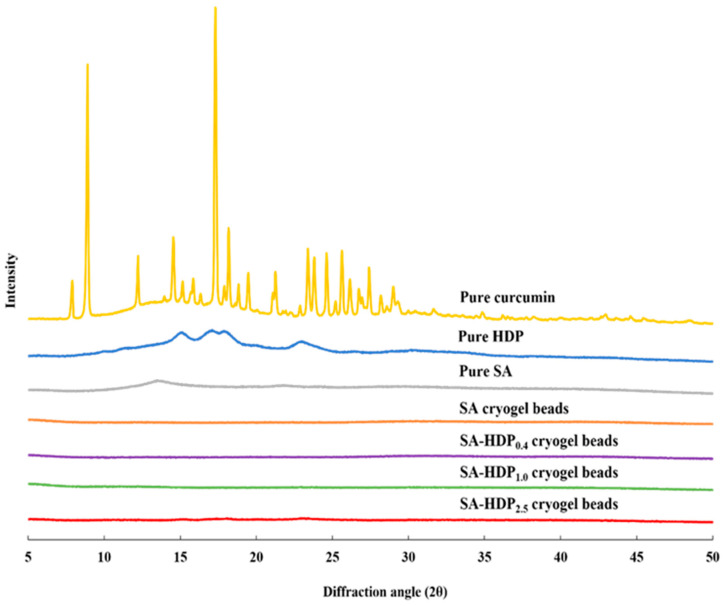
X-ray diffraction (XRD) patterns of pure curcumin, pure hydroxypropyl distarch phosphate (HDP), pure sodium alginate (SA), and SA-based cryogel beads filled with different concentrations of HDP. SA cryogel beads, SA-HDP_0.4_ cryogel beads, SA-HDP_1.0_ cryogel beads, and SA-HDP_2.5_ cryogel beads represent cryogel beads filled with different concentrations of HDP (0, 0.4, 1.0, and 2.5%, respectively).

Figure 2 illustrates the XRD patterns of pure curcumin, pure HDP, pure SA, SA cryogel beads, SA-HDP_0.4_ cryogel beads, SA-HDP_1.0_ cryogel beads, and SA-HDP_2.5_ cryogel beads. Curcumin showed a highly crystalline structure with sharp characteristic peaks at 8°, 12°, 14°, 17°, 23°, 24° and 25° [27]. HDP had an A type diffraction pattern and semi-crystalline structure with characteristic peaks at 15°, 17°, and 23° [28]. SA had a characteristic peak at 13° [29]. In the present study, the sharp characteristic peaks (8°, 12°, 14°, 17°, 23°, 24° and 25°) of curcumin disappeared in all cryogel beads. A similar result was observed when curcumin was encapsulated in polylactide-co-glycolide nanoparticles, in which no characteristic peaks of curcumin were shown because polylactide-co-glycolide nanoparticles formed an amorphous complex with intermolecular interaction in the matrix [30]. Liu et al. [6] reported a similar result in that the sharp crystalline peaks of curcumin disappeared in the lotus root amylopectin-chitosan complex hydrogel due to the formation of an amorphous complex between curcumin and the lotus root amylopectin-chitosan matrix. In addition, Acevedo-Guevara et al. [31] reported that encapsulated curcumin existed in an amorphous complex rather than a crystalline structure during encapsulation of curcumin in the banana starch nanoparticles. As a result, it is indicated in the present study that the disappearance of the characteristic peaks for curcumin was ascribed to the formation of amorphous complex with intermolecular interaction occurring within the matrix of SA and HDP.

### 3.3. Differential Scanning Calorimetry (DSC) Analysis

**Figure 3:**
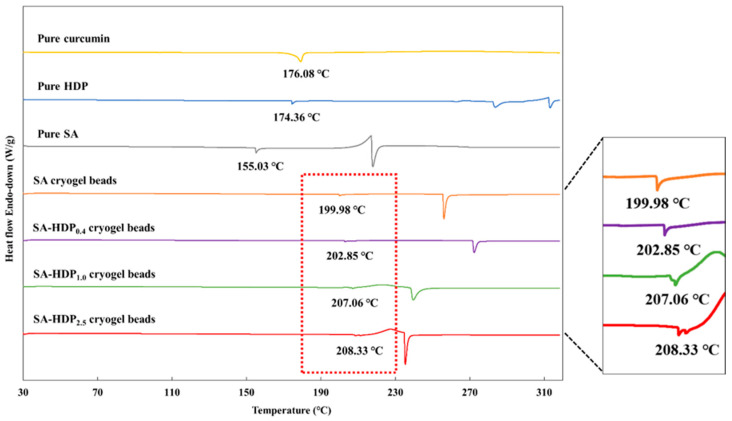
Differential scanning calorimetry (DSC) analyses of pure curcumin, pure hydroxypropyl distarch phosphate (HDP), pure sodium alginate (SA), and SA-based cryogel beads filled with different concentrations of HDP. SA cryogel beads, SA-HDP_0.4_ cryogel beads, SA-HDP_1.0_ cryogel beads, and SA-HDP_2.5_ cryogel beads represent cryogel beads filled with different concentrations of HDP (0, 0.4, 1.0, and 2.5%, respectively).

DSC was conducted to study the thermal properties of wall materials and SA-based cryogel beads filled with different concentrations of HDP. Figure 3 shows DSC thermograms of pure curcumin, pure HDP, pure SA, SA cryogel beads, SA-HDP_0.4_ cryogel beads, SA-HDP_1.0_ cryogel beads, and SA-HDP_2.5_ cryogel beads. The endothermic peak of pure curcumin was found at 176.08 °C, which is the melting temperature (T_m_) of the crystalline form. Patel et al. [27] reported that the sharp melting peak of curcumin was exhibited at 175 °C. The DSC thermograms of pure HDP showed the endothermic peak at 174.36 °C. Pure SA exhibited the endothermic peak at 155.03 °C, which is related to the melting of the crystal form of alginate [32,33]. The T_m_ of SA-based cryogel beads was shifted from 199.98 °C to 208.33 °C as HDP concentration was increased from 0% to 2.5%, respectively. According to Hosseini et al. [34], T_m_ was shifted to a higher temperature by adding resistant starch to alginate microparticles. López Córdoba et al. [18] reported that adding corn starch to calcium-crosslinked SA hydrogel beads improved thermal stability by shifting T_m_ from 198 °C to 201 °C. In addition, the endothermic peak of curcumin was not exhibited in SA-based cryogel beads, because curcumin formed an amorphous structure through the intermolecular interaction with SA and HDP. Therefore, these results indicated that adding HDP as a filler to SA-based cryogel beads improved the thermal stability and confirmed the encapsulation of curcumin.

### 3.4. Fourier Transform Infrared (FT-IR) Spectroscopy Analysis

**Figure 4:**
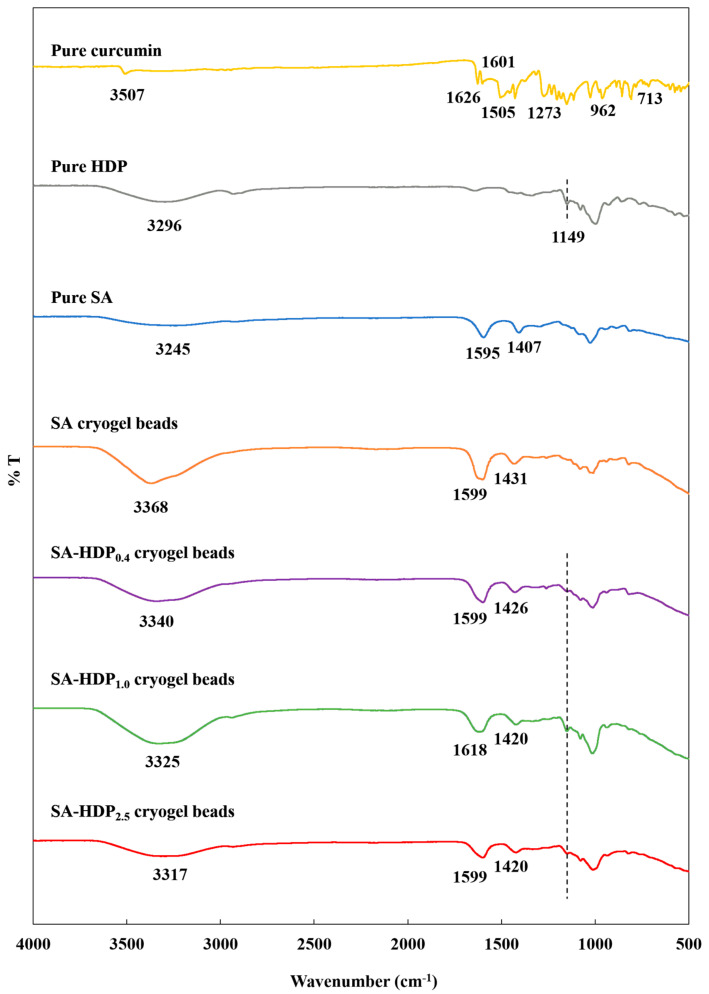
FT−IR spectrum of pure hydroxypropyl distarch phosphate (HDP), pure sodium alginate (SA), pure curcumin, and SA-based cryogel beads filled with different concentrations of HDP. SA cryogel beads, SA-HDP_0.4_ cryogel beads, SA-HDP_1.0_ cryogel beads, and SA-HDP_2.5_ cryogel beads represent cryogel beads filled with different concentrations of HDP (0, 0.4, 1.0, and 2.5%, respectively).

Figure 4 shows the FT-IR spectrum of pure curcumin, pure sodium alginate (SA), pure hydroxypropyl distarch phosphate (HDP), SA cryogel beads, SA-HDP_0.4_ cryogel beads, SA-HDP_1.0_ cryogel beads, and SA-HDP_2.5_ cryogel beads. The characteristic peaks for pure curcumin showed at 3507 cm^−1^, and 3015 cm^−1^, which corresponded to phenolic O-H stretching vibrations, and C-H stretching vibrations, respectively [35]. The sharp peaks at 1626 cm^−1^ and 1601 cm^−1^ were attributed to C=C and C=O vibrations, respectively [36]. The peak at 1601 cm^−1^ was related to the stretching vibration of the benzene ring of curcumin. The peaks at 1505 cm^−1^ and 1273 cm^−1^ corresponded to C=O vibration and enol-C-O structure of curcumin, respectively [36]. The absorption bands at 1026 cm^−1^ and 855 cm^−1^ were associated to C-O-C stretching, and the peak at 962 cm^−1^ was assigned to benzoate trans-CH vibration of curcumin. The peak at 713 cm^−1^ was attributed to the cis-CH vibration of the aromatic ring [36].

For the FT-IR spectrum of SA, O-H stretching vibration showed at 3245 cm^−1^ and the asymmetric stretching of carboxyl (-COO^−^) group and symmetric stretching of the COO^−^ group were found at 1595 cm^−1^ and 1407 cm^−1^, respectively [37]. Furthermore, there were multiple vibration peaks related to the polysaccharides at 900–1350 cm^−1^ [38].

As shown in the FT-IR spectrum of HDP, a characteristic broad peak at 3296 cm^−1^ was attributed to O-H stretching vibrations, and a peak at 2931 cm^−1^ was associated to C-H symmetric stretching vibrations [28]. The peak at 1149 cm^−1^ was assigned to the C-O-C ether bond in hydroxypropylated starch by propylene oxide and the glycosidic bond polymerization of glucose in HDP at the same time [39]. Furthermore, the peaks at 1077 cm^−1^ and 927 cm^−1^ were attributed to C-O-P and O-P-O of the phosphate group, which is formed through esterification [40]. According to Deeyai et al. [41], the bands at 900–1200 cm^−1^ were related to C-O-C stretching vibrations in the glycosidic bonds of glucose rings.

No characteristic peaks of curcumin in all cryogel beads were observed, which indicated the successful encapsulation in all cryogel beads matrices. According to Acevedo-Guevara et al. [31], overlapping of the curcumin characteristic peaks in the starch nanoparticles suggested that curcumin did not exist as free molecules. Furthermore, Mohammadian et al. [42] reported that the disappeared peaks of curcumin after encapsulation in the whey protein microgels were due to the incorporation of curcumin into a delivery system.

The peaks at 1595 and 1407 cm^−1^ of SA were shifted to higher wavenumbers (1599–1618 cm^−1^, 1420–1431 cm^−1^), respectively, suggesting that the-COO^−^ of SA were ionically crosslinked with CaCl_2_ (Ca^2+^). Hua et al. [43] reported that crosslinking between -COO^−^ of SA and Ca^2+^ shifted the asymmetric stretching of -COO^−^ and asymmetric stretching of -COO^−^ to higher wavenumbers.

The formation of hydrogen bonds is an important factor affecting the structural properties of the cryogel beads. Compared to the SA cryogel beads (3368 cm^−1^), the peaks at O-H stretching vibrations of SA-HDP_0.4_ cryogel beads, SA-HDP_1.0_ cryogel beads, and SA-HDP_2.5_ cryogel beads were shifted to lower wavenumbers, which were 3340 cm^−1^, 3337 cm^−1^, and 3317 cm^−1^, respectively. These results demonstrated that the new intermolecular hydrogen bonds were between the hydroxyl groups in SA, HDP, and curcumin. According to Wang et al. [44], the peak of O-H stretching was shifted from 3394 to 3381 cm^−1^, indicating the formation of intermolecular hydrogen bonds by adding cellulose nanocrystals in the deep eutectic solvent-hydrogel. Feng et al. [45] reported that the O-H stretching peak of sodium alginate/poly (vinyl alcohol)/*Lactobacillus plantarum* fiber mat was shifted to a lower wavenumber, indicating that more hydrogen bonds were formed between SA and PVA. In addition, the O-H stretching peak of the alginate microparticle and alginate-resistant starch microparticle was shifted from 3430 cm^−1^ to 3411 cm^−1^, which was ascribed to the increasing of hydrogen bonds between the alginate and resistant starch [34].

For the FT-IR spectrum of SA-HDP_0.4_ cryogel beads, SA-HDP_1.0_ cryogel beads, and SA-HDP_2.5_ cryogel beads, the regions between 900–1200 cm^−1^ (C-O-C stretching vibrations in glycosidic bonds in glucose rings) were changed after HDP was added. In particular, the peak at 1149 cm^−1^ (C-O-C ether bond of chemically modified starch) of HDP appeared in the SA-based cryogel beads filled with HDP but the peak was not observed in SA cryogel beads. These results indicated that HDP was successfully added into the SA cryogel beads to fill the pores. According to Lozano-Vazquez et al. [13], the peaks of C-O-C stretching vibrations for tapioca modified starch was found in the alginate beads, when tapioca modified starch was used as a filler in the alginate beads.

Therefore, these results suggested that the cryogel beads produced in this study improved intermolecular hydrogen bonds between wall materials and core materials, ionic crosslinking between SA-Ca^2+^ was well formed, and that the HDP granule acted as a filler.

### 3.5. Encapsulation Efficiency of Curcumin

The encapsulation efficiency is an important indicator for measuring and evaluating the content of bioactive compounds in a delivery system [6]. The encapsulation efficiency of curcumin in the cryogel beads filled with different concentrations of HDP is shown in Table 1. The encapsulation efficiency of curcumin in cryogel beads was significantly increased from 31.95% to 76.66% with increasing the concentration of HDP from 0% to 2.5%, respectively. According to Chan et al. [25], the relative porosity of the lyophilized calcium-alginate beads was about 70%, which was three times higher than that of the cryogel beads added with starch at a concentration of 600 g/L. Stojanovic et al. [46] reported that the low encapsulation efficiency of calcium alginate hydrogel beads containing thyme extract is because the bioactive compounds easily diffused into the gelling solution during the encapsulation process. However, they reported that the encapsulation efficiency was increased from 51% to 79% by adding inulin as a filler to calcium alginate hydrogel beads containing thyme extract. Furthermore, Li et al. [19] reported that the encapsulation efficiency of tea polyphenols was increased from 38.51% to 48.56% when gum arabic was added to the Ca(II)-alginate-based beads. Lozano-Vazquez et al. [13] also found that the addition of modified tapioca starch to alginate hydrogel beads remarkably increased the encapsulation efficiency because modified tapioca starch served as a filler and prevented the diffusion of chlorogenic acid. Therefore, in the present study, the lowest encapsulation efficiency of SA cryogel beads can be related to the diffusional transport of curcumin stock solution during the crosslinking in the CaCl_2_ solution. However, the encapsulation efficiency was significantly increased when HDP was added to the SA-based cryogel beads. This finding can be associated with the function of HDP as a filler to fill the remaining spaces during the crosslinking process. In other words, HDP formed cryogel beads with a denser internal structure and reduced the diffusional transport of the curcumin.

As previously mentioned in SEM images (Figure 1), SA-based cryogel beads filled with HDP had a denser internal structure. In addition, the result of FT-IR (Figure 4), increasing hydrogen bonds (O-H stretching vibrations shifted to lower wavenumber) and the presence of HDP (C-O-C ether bond) in SA-based cryogel beads filled with HDP improved the structural properties. Therefore, in the present study, HDP can improve the structural properties of cryogel beads by effectively acting as a filler, and consequently can increase the encapsulation efficiency of curcumin.

### 3.6. Swelling Ratio

**Figure 5:**
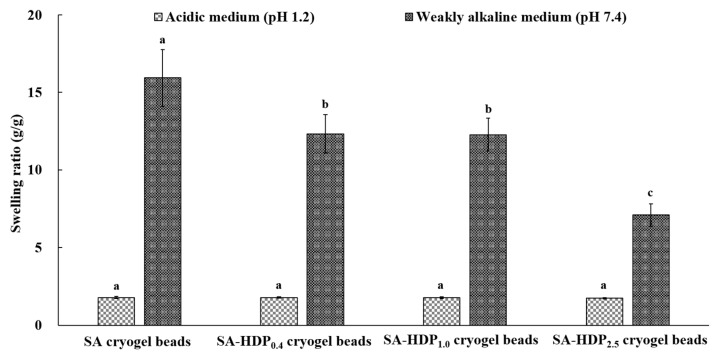
Swelling ratio of sodium alginate (SA)-based cryogel beads filled with different concentrations of hydroxypropyl distarch phosphate (HDP) under different pH conditions. SA cryogel beads, SA-HDP_0.4_ cryogel beads, SA-HDP_1.0_ cryogel beads, and SA-HDP_2.5_ cryogel beads represent cryogel beads filled with different concentrations of HDP (0, 0.4, 1.0 and 2.5%, respectively). Values with different letters within the same column differ significantly (*p* < 0.05).

Figure 5 shows the swelling ratio of cryogel beads in acidic medium (pH 1.2) and alkaline medium (pH 7.4). In acidic medium, the swelling ratio of all cryogel beads showed no significant difference. However, the swelling ratio of all cryogel beads in acidic medium was lower than that in alkaline medium. Because the pKa of carboxyl groups in SA is approximately 3.2, the protonation of carboxyl groups can occur at pH 1.2 (<pKa ~3.2). Therefore, the –COOH can be the predominate form in the cryogel beads, which results in a lower electrostatic repulsive force and preventing the cryogel beads from swelling. According to Dafe et al. [47], the swelling ratio of pectin/starch hydrogels at pH 1.2 was lower than at pH 7.4, because the carboxyl group of pectin was protonated and the electrostatic repulsive force was reduced. In alkaline medium, the swelling ratios of SA cryogel beads, SA-HDP_0.4_ cryogel beads, SA-HDP_1.0_ cryogel beads, and SA-HDP_2.5_ cryogel beads were 15.94, 12.33, 12.27, and 7.09 (g/g), respectively. The swelling ratio was significantly decreased with increasing HDP concentrations. Since the pH 7.4 (>pKa ~3.2) was higher than pKa value of the carboxyl group, it can be converted to an anionic carboxyl group (-COO^−^), and the swelling was promoted by increasing the electrostatic repulsive force between the -COO^−^ [48]. According to Wang et al. [44], as the concentration of resistant starch in the β-cyclodextrin/oxidized gellan gum/resistant starch hydrogel beads loaded with resveratrol was increased, the electrostatic repulsive force between the -COO^−^ carboxyl groups of the oxidized gellan gum at pH 7.0 was weakened, resulting in a reduced swelling ratio. Therefore, in the present study, the increasing concentrations of HDP weakened the electrostatic repulsive force between anionic -COO^−^ of the SA-based network, consequently, decreasing the swelling ratio. These results indicated that SA-based cryogel beads can be pH-sensitive in the different pH solutions and HDP can protect the structural deformation of SA-based cryogel beads under alkaline conditions.

### 3.7. In Vitro Gastrointestinal Tract Release Study

**Figure 6:**
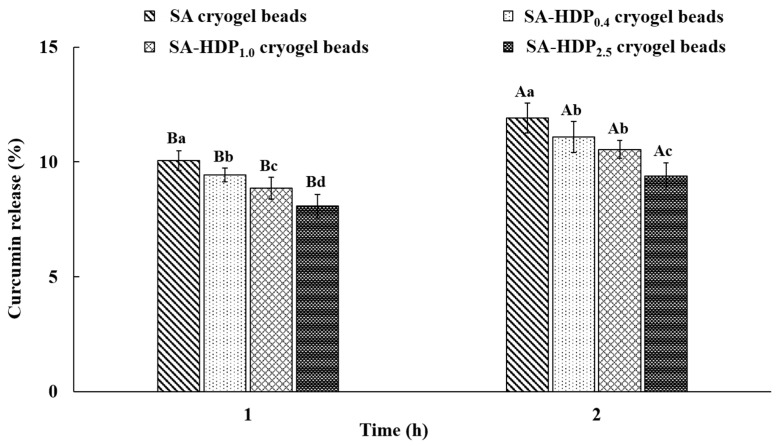
In vitro gastrointestinal tract release properties of sodium alginate (SA)-based cryogel beads filled with different concentrations of hydroxypropyl distarch phosphate (HDP) in simulated gastric fluid (SGF). SA cryogel beads, SA-HDP_0.4_ cryogel beads, SA-HDP_1.0_ cryogel beads, and SA-HDP_2.5_ cryogel beads represent cryogel beads filled with different concentrations of HDP (0, 0.4, 1.0 and 2.5%, respectively). Small letters show significant differences between treatment at the same time (*p* < 0.05), and capital letters show significant differences between times for the same treatment (*p* < 0.05).

**Figure 7:**
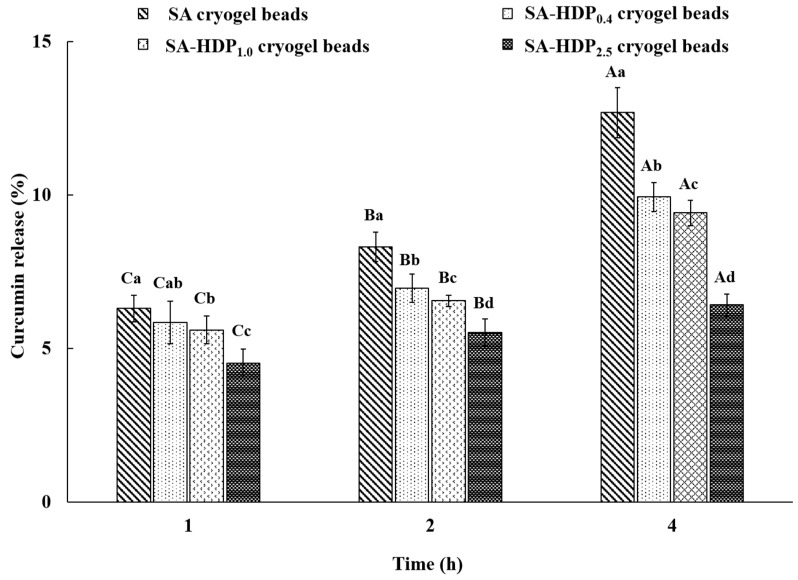
In vitro gastrointestinal tract release properties of sodium alginate (SA)-based cryogel beads filled with different concentrations of hydroxypropyl distarch phosphate (HDP) in simulated intestinal fluid (SIF). SA cryogel beads, SA-HDP_0.4_ cryogel beads, SA-HDP_1.0_ cryogel beads, and SA-HDP_2.5_ cryogel beads represent cryogel beads filled with different concentrations of HDP (0, 0.4, 1.0 and 2.5%, respectively). Small letters show significant differences between treatments at the same time (*p* < 0.05), and capital letters show significant differences between times for the same treatment (*p* < 0.05).

The in vitro gastrointestinal tract release study of cryogel beads was carried out to investigate the controlled-release of the core material (curcumin) in simulated gastric fluid (SGF, pH 1.2) and simulated intestinal fluid (SIF, pH 7.4). The in vitro gastrointestinal tract release rate of curcumin in SGF and SIF are shown in Figure 6 and Figure 7, respectively. The release rate of curcumin in cryogel beads was decreased after 2 h in SGF and 4 h in SIF as the HDP concentration was increased.

In general, it is noted that curcumin is stable in an acidic environment, but is unstable in an alkaline environment, which led to the rapid color change [49,50]. Curcumin passes through the stomach without the chemical or structural modification due to its resistance to acidic environments. However, the half-life of curcumin is 10 min in phosphate buffer at pH 7.4 (SIF condition), and the oral bioavailability of curcumin is poor due to low intestinal absorption [8,9]. In addition, a small amount of absorbed curcumin undergoes rapid metabolism in the liver, and is ultimately excreted in urine and feces without being used for most tissues or cells [10]. Therefore, it is suggested in the present study that the curcumin can pass through the gastrointestinal tract and then can safely reach to the colon and be metabolized by colonic microorganisms for increasing the physiological benefits.

After 2 h in SGF, the release rate of curcumin in cryogel beads was significantly decreased from 11.92% to 9.38%, as HDP concentration was increased from 0% to 2.5%, respectively. According to Belščak-Cvitanović et al. [51], the rapid release of polyphenols from the porous alginate network in SGF (pH 2.0) was due to the release of loosely bound or adsorbed polyphenols on the surface in alginate microparticle beads. As described in SEM images (Figure 1) previously, SA cryogel beads showed cracks on the surface and a less dense internal structure, which can lead to the release of curcumin. When cracks presented on the surface, SGF could be diffused into the cryogel beads, which can cause the rapid release of curcumin. Li et al. [19] reported that adding a filler (inulin, arabic gum, and chitosan) to the sodium alginate-based hydrogel beads inhibited the release of tea polyphenol in SGF. Furthermore, Bušić et al. [14] reported that adding fillers (e.g., whey protein isolate, cocoa powder, and carob) to alginate hydrogel beads reduced the diffusion of SGF and the release rate of dandelion polyphenols due to the reinforced structure formed by the addition of fillers. Therefore, in the present study, it was indicated that the addition of HDP acted as a structural support, preventing the diffusion of SGF into the internal structure in cryogel beads and reducing the release rate of curcumin.

After 4 h in SIF, the release rate of curcumin in SA cryogel beads, SA-HDP_0.4_ cryogel beads, SA-HDP_1.0_ cryogel beads, and SA-HDP_2.5_ cryogel beads were 12.68%, 9.93%, 9.41%, and 6.41%, respectively. At pH 7.0, the uncrosslinked or disrupted carboxyl groups of alginate were deprotonated to absorb a large amount of water and consequently to loosen the structure due to swelling. Accordingly, in this study, in SIF (pH 7.4 > pKa), the carboxyl groups of sodium alginate which were not crosslinked by Ca^2+^ ions are deprotonated to form anionic -COO^−^, and greater swelling occurred due to increasing electrostatic repulsive forces. This phenomenon was one of the major factors for the release of curcumin in SA cryogel beads in the SIF. However, the release rate of curcumin in cryogel beads was significantly reduced with increasing concentrations of HDP in the SIF, because the presence of HDP reduced the electrostatic repulsive force that occurred between the -COO^−^ in the SA-based cryogel beads network [47]. Therefore, increasing the HDP concentration reduced the swelling of the cryogel beads in the SIF, resulting in a less loose structure. These results are consistent with the swelling ratio in this study (Figure 5). Wang et al. [44] reported that the release rate of resveratrol was decreased with the increase in the concentration of resistant starch in SIF. Furthermore, it was suggested in the present study that HDP (resistant starch type 4) was not able to be broken down by the attack of digestive enzymes (pancreatin) under SIF conditions and fermented by colonic microorganisms in the colon [26,52]. Therefore, the addition of HDP to SA-based cryogel beads decreased the swelling behavior by reducing the electrostatic repulsive force in SIF and prevented the access of pancreatin. Consequently, it was indicated that HDP served as a structural support for SA-based cryogel beads under harsh conditions.

It was possible to effectively protect the curcumin and control the release rate of curcumin in both of SGF and SIF, thereby overcoming the disadvantage of the poor oral bioavailability of curcumin. In fact, when it passed through the small intestine after ingestion, the curcumin remaining in the cryogel beads can reach the colon and can be metabolized by colonic microorganisms. Biotransformation and degradation of curcumin occurs by many colonic microorganisms such as *Lactobacillus*, *Bifidobacterium*, and *E. coli*, which promotes beneficial bacterial strains and improves intestinal barrier function [53]. According to Zam [12], tetrahydrocurcumin (THC), dihydroferulic acid (DFA), and 1-(4-hydroxy-3-methoxyphenyl)-2-propanol were detected as the main metabolites of curcumin in the human fecal fermentation cultures. When curcumin is metabolized, it can provide health benefits because of its antioxidant [6], anti-inflammatory [7], and immunomodulatory properties [5]. These results demonstrated that cryogel beads were a promising delivery system that can control the release rate of curcumin from the gastrointestinal tract and safely deliver curcumin to the colon for health benefits.

## 4. Conclusions

In the present study, SA-based cryogel beads filled with different concentrations of HDP were successfully prepared as a curcumin delivery system. An SEM analysis revealed that HDP served as a filler to form spherical cryogel beads with a denser internal structure. According to XRD analysis, curcumin was successfully encapsulated in the cryogel beads. New ionic crosslinking between carboxyl groups (-COO^−^) of SA and calcium ions (Ca^2+^) and new intermolecular hydrogen bonds between hydroxyl groups in SA, HDP, and curcumin were found in the FT-IR spectrum. A DSC analysis indicated that adding HDP to SA-based cryogel beads can improve thermal stability. The encapsulation efficiency of curcumin was increased by increasing the concentrations of HDP. The release rate of curcumin from SA-based cryogel beads in the simulated gastrointestinal tract was decreased with increasing the concentrations of HDP, indicating that SA-based cryogel beads filled with HDP have a protective effect for curcumin. Not only the higher encapsulation efficiency but also the lower release rate of curcumin in SGF and SIF for the SA-based cryogel beads filled with HDP can be associated to the compact and reinforced SA-based structure filled with HDP. These observations clearly indicate that SA-based cryogel beads filled with HDP can be suitable for the colon-targeted delivery system without the premature release of curcumin in the stomach and small intestine. In addition, SA-based cryogel beads filled with HDP can increase bioavailability by allowing curcumin to reach the colon and can be metabolized by colonic microorganisms. In conclusion, this study introduced the novel cryogel beads made up with SA and HDP as an oral delivery carrier of curcumin in the food industry, and HDP as a good filler for wall material can be further used to develop various delivery systems.

## Figures and Tables

**Table 1:** Encapsulation efficiency of curcumin in sodium alginate (SA)-based cryogel beads filled with different concentrations of hydroxypropyl distarch phosphate (HDP).

Samples ^(1)^	Encapsulation Efficiency (%)
SA cryogel beads	31.95 ± 0.14 ^d(2)^
SA-HDP_0.4_ cryogel beads	45.61 ± 0.81 ^c^
SA-HDP_1.0_ cryogel beads	56.52 ± 0.69 ^b^
SA-HDP_2.5_ cryogel beads	76.66 ± 0.98 ^a^

^(1)^ SA cryogel beads, SA-HDP_0.4_ cryogel beads, SA-HDP_1.0_ cryogel beads, and SA-HDP_2.5_ cryogel beads represent cryogel beads filled with different concentrations of HDP (0, 0.4, 1.0 and 2.5%, respectively). ^(2)^ Values with different letters within the same column differ significantly (*p* < 0.05).

## Data Availability

Not applicable.

## Sample Availability

Samples of the compounds are not available from the authors.
